# Averting Obesity and Type 2 Diabetes in India through Sugar-Sweetened Beverage Taxation: An Economic-Epidemiologic Modeling Study

**DOI:** 10.1371/journal.pmed.1001582

**Published:** 2014-01-07

**Authors:** Sanjay Basu, Sukumar Vellakkal, Sutapa Agrawal, David Stuckler, Barry Popkin, Shah Ebrahim

**Affiliations:** 1 Prevention Research Center; Centers for Health Policy, Primary Care and Outcomes Research; Center on Poverty and Inequality; and Cardiovascular Institute, Stanford University, Stanford, California, United States of America; 2 Department of Public Health and Policy, London School of Hygiene & Tropical Medicine, London, United Kingdom; 3 South Asia Network for Chronic Disease, Public Health Foundation of India, New Delhi, India; 4 Department of Sociology, Oxford University, Oxford, United Kingdom; 5 School of Public Health, University of North Carolina at Chapel Hill and the Carolina Population Center, Chapel Hill, North Carolina, United States of America; 6 Department of Non-Communicable Disease Epidemiology, London School of Hygiene & Tropical Medicine, London, United Kingdom; University of Otago, Wellington, New Zealand

## Abstract

**Background:**

Taxing sugar-sweetened beverages (SSBs) has been proposed in high-income countries to reduce obesity and type 2 diabetes. We sought to estimate the potential health effects of such a fiscal strategy in the middle-income country of India, where there is heterogeneity in SSB consumption, patterns of substitution between SSBs and other beverages after tax increases, and vast differences in chronic disease risk within the population.

**Methods and Findings:**

Using consumption and price variations data from a nationally representative survey of 100,855 Indian households, we first calculated how changes in SSB price alter per capita consumption of SSBs and substitution with other beverages. We then incorporated SSB sales trends, body mass index (BMI), and diabetes incidence data stratified by age, sex, income, and urban/rural residence into a validated microsimulation of caloric consumption, glycemic load, overweight/obesity prevalence, and type 2 diabetes incidence among Indian subpopulations facing a 20% SSB excise tax. The 20% SSB tax was anticipated to reduce overweight and obesity prevalence by 3.0% (95% CI 1.6%–5.9%) and type 2 diabetes incidence by 1.6% (95% CI 1.2%–1.9%) among various Indian subpopulations over the period 2014–2023, if SSB consumption continued to increase linearly in accordance with secular trends. However, acceleration in SSB consumption trends consistent with industry marketing models would be expected to increase the impact efficacy of taxation, averting 4.2% of prevalent overweight/obesity (95% CI 2.5–10.0%) and 2.5% (95% CI 1.0–2.8%) of incident type 2 diabetes from 2014–2023. Given current consumption and BMI distributions, our results suggest the largest relative effect would be expected among young rural men, refuting our a priori hypothesis that urban populations would be isolated beneficiaries of SSB taxation. Key limitations of this estimation approach include the assumption that consumer expenditure behavior from prior years, captured in price elasticities, will reflect future behavior among consumers, and potential underreporting of consumption in dietary recall data used to inform our calculations.

**Conclusion:**

Sustained SSB taxation at a high tax rate could mitigate rising obesity and type 2 diabetes in India among both urban and rural subpopulations.

*Please see later in the article for the Editors' Summary*

## Abstract 2

In this modeling study, Sanjay Basu and colleagues estimate the potential health effects of a sugar-sweetened beverage taxation among various sub-populations in India over the period 2014 to 2023.

*Please see later in the article for the Editors' Summary*

## Editors' Summary

**Background:**

Non-communicable diseases (NCDs) and obesity (excessive body mass) are major threats to global health. Each year NCDs kill 36 million people (including 29 million people in low- and middle-income countries), thereby accounting for nearly two-thirds of the world's annual deaths. Cardiovascular diseases, cancers, respiratory diseases, and diabetes (a condition characterized by raised blood sugar levels) are responsible for most NCD-related deaths. Worldwide, diabetes alone affects about 360 million people and causes nearly 5 million deaths annually. And the number of people affected by NCDs is likely to rise over the next few decades. It is estimated, for example, that 101.2 million people in India will have diabetes by 2030, nearly double the current number. In Asia and other low- and middle-income countries overweight as well as obesity represent a risk factor for NCDs and the global prevalence of obesity (the proportion of the world's population that is obese) has nearly doubled since 1980. Worldwide, around 0.5 billion people are now classified as obese and about 1.5 billion more overweight. That is, they have a body mass index (BMI) of 30 kg/m^2^ or more (25–30 for overweight); BMI is calculated by dividing a person's weight in kilograms by the square of their height in meters. In India individuals with a BMI of 25 or more (overweight/obese) are at very high risk of diabetes.

**Why Was This Study Done?:**

The consumption of sugar-sweetened beverages (SSBs, soft drinks sweetened with cane sugar or other caloric sweeteners) is a major risk factor for overweight/obesity and, independent of total energy consumption and BMI, for type 2 diabetes (the commonest form of diabetes). In high-income countries, SSB taxation has been proposed as a way to lower the risk of obesity and type 2 diabetes, however it is unknown if this approach will work in low- and middle-income countries. Here, in an economic-epidemiologic modeling study, researchers estimate the potential health effects of SSB taxation in India, a middle-income country in which total SSB consumption is rapidly increasing, but where SSB consumption and chronic disease risk vary greatly within the population and where people are likely to turn to other sugar-rich beverages (for example, fresh fruit juices) if SSBs are taxed.

**What Did the Researchers Do and Find?:**

The researchers used survey data relating SSB consumption to price variations to calculate how changes in the price of SSBs affect the demand for SSBs (own-price elasticity) and for other beverages (cross-price elasticity) in India. They combined these elasticities and data on SSB sales trends, BMIs, and diabetes incidence (the frequency of new diabetes cases) into a mathematical microsimulation model to estimate the effect of a 20% tax on SSBs on caloric (energy) consumption, glycemic load (an estimate of how much a food or drink raises blood sugar levels after consumption; low glycemic load diets lower diabetes risk), the prevalence of overweight/obesity, and the incidence of diabetes among Indian subpopulations. According to the model, if SSB sales continue to increase at the current rate, compared to no tax, a 20% SSB tax would reduce overweight/obesity across India by 3.0% and the incidence of type 2 diabetes by 1.6% over the period 2014–2023. In absolute figures, a 20% SSB tax would avert 11.2 million cases of overweight/obesity and 400,000 cases of type 2 diabetes between 2014 and 2023. Notably, if SSB sales increase more steeply as predicted by drinks industry marketing models, the tax would avert 15.8 million cases of overweight/obesity and 600,000 cases of diabetes. Finally, the model predicted that the largest relative effect of an SSB tax would be among young men in rural areas.

**What Do These Findings Mean?:**

The accuracy of these findings is likely to be affected by the assumptions incorporated in the model and by the data fed into it. In particular, the accuracy of the estimates of the health effects of a 20% tax on SSBs is limited by the assumption that future consumer behavior will reflect historic behavior and by potential underreporting of SSB consumption in surveys. Nevertheless, these findings suggest that a sustained high rate of tax on SSBs could mitigate the rising prevalence of obesity and the rising incidence of diabetes in India in both urban and rural populations by affecting both caloric intake and glycemic load. Thus, SSB taxation might be a way to control obesity and diabetes in India and other low- and middle-income countries where, to date, large-scale interventions designed to address these threats to global health have had no sustained effects.

**Additional Information:**

Please access these websites via the online version of this summary at http://dx.doi.org/10.1371/journal.pmed.1001582.

- The World Health Organization provides information about non-communicable diseases, obesity, and diabetes around the world (in several languages)
- The US Centers for Disease Control and Prevention provides information on non-communicable diseases around the world and on overweight and obesity and diabetes (including some information in Spanish)
- The US National Diabetes Information Clearinghouse provides information about diabetes for patients, health-care professionals, and the general public, including detailed information on weight control (in English and Spanish)
- The UK National Health Service Choices website provides information for patients and carers about type 2 diabetes and about obesity; it includes personal stories about diabetes and about obesity
- MedlinePlus provides links to further resources and advice about diabetes and diabetes prevention and about obesity (in English and Spanish)
- A 2012 Policy brief from the Yale Rudd Center for food policy and obesity provides information about SSB taxes

## Introduction

**Figure 1:**
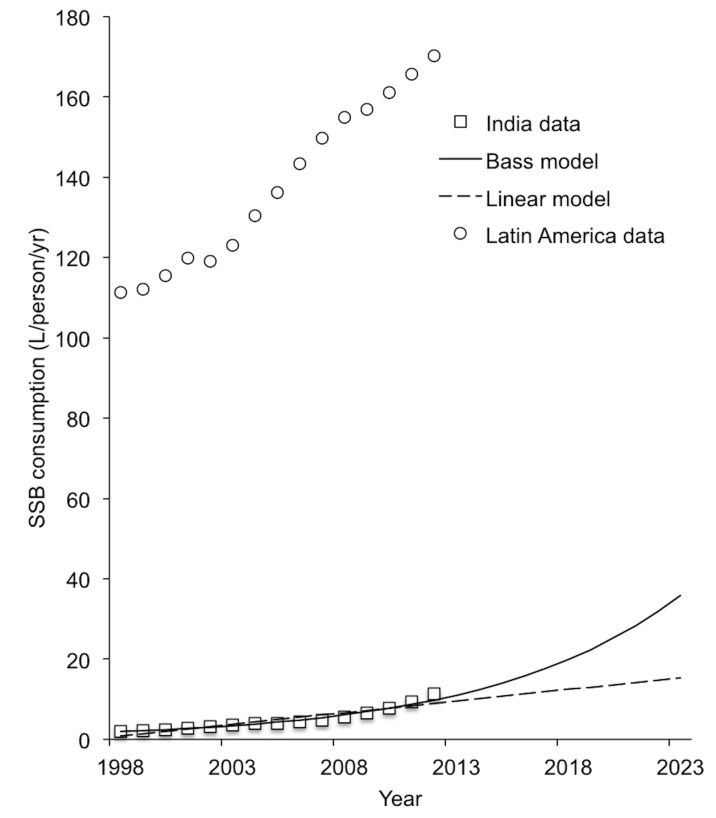
SSB consumption in liters per capita per year in India, 1998–2012 [9]. The Latin American average consumption is plotted for comparison, reflecting the population-weighted average per capita consumption from 13 countries (Argentina, Bolivia, Brazil, Chile, Colombia, Costa Rica, Dominican Republic, Ecuador, Guatemala, Mexico, Peru, Uruguay, and Venezuela).

Sugar-sweetened beverage (SSB) consumption is established as a major risk factor for overweight and obesity, as well as an array of cardio-metabolic conditions, especially type 2 diabetes [1],[2]. The individual risk of type 2 diabetes attributable to SSB consumption remains statistically significant after adjustment for total energy consumption and body mass index (BMI) [3],[4]. While taxes on SSBs have been proposed in high-income countries to lower obesity and type 2 diabetes risks given limited success from other population measures and individual-level interventions [5], recent assessments reveal a majority of SSB sales now occur outside the US and Europe, where marketing efforts appear most focused [6]–[8]. SSB sales in India, for example, have increased by 13% year-on-year since 1998, exceeding 11 liters per capita per year (Figure 1) [9]. At the population level, the acceleration of SSB consumption among middle-income country populations has been statistically associated with increased obesity, overweight, and type 2 diabetes prevalence rates, independent of concurrent changes in other caloric consumption, physical inactivity, and aging [6],[10],[11].

While econometric and modeling studies suggest the potential effectiveness of large (e.g., penny-per-ounce, or 20%–25%) but not smaller excise taxes on SSBs in the United States [12]–[15], and UK [16], a key unknown is whether such fiscal strategies will be wise to implement in middle-income nations like India and China, where several aspects of SSB consumption and disease risk are uniquely different from Western populations [17]. Asian countries' populations appear to be internally heterogeneous in their “nutrition transition” towards Western dietary patterns high in salt, sugar, and fat content [17],[18]. This implies that nationwide taxation may be perverse if benefits accrue among only select populations while monetary penalties apply universally, especially if the tax burden but not the tax benefit falls disproportionately on the poor. In India, processed foods make up a substantial portion of dietary consumption overall, but with marked variations between men and women and among age groups, income classes, and urban versus rural populations [19],[20]. Type 2 diabetes is similarly more prevalent among urban, higher-income men, but lower-income groups and women increasingly face a heightened burden of obesity and low diagnosis rates associated with poor health care access [21]–[23]. Furthermore, the beverages that people consume apart from SSBs also contain high caloric and glycemic loads in India and much of Asia [6], indicating that if a tax were to induce substitutions from SSBs to other beverages, the net health impacts on obesity and type 2 diabetes may be limited and, possibly, perverse [24].

We sought to characterize the influence of an SSB tax on overweight, obesity, and type 2 diabetes trajectories among multiple demographic groups in India. To perform the analysis, we first used a standard microeconomic approach to calculate how changes in SSB price relate to changes in SSB consumption (“own-price elasticity”) and substitution of SSBs for other beverages (“cross-price elasticity”), using per capita consumption and price variations data from a nationally representative household survey [25]. We then estimated how changes in overall calories and glycemic load induced by a 20% excise tax on SSBs would be expected to alter overweight, obesity, and type 2 diabetes incidence over the period 2014–2024. We chose the 20% rate for comparability against tax simulations in Western populations, where a penny-per-ounce tax amounts to an ∼20%–25% price increase [15]; the 20% change is also within the 35% SSB price variation range in the survey data employed for our assessment. We nevertheless varied the tax rate from 10% to 30% in sensitivity analyses to explore alternative forecasts. We constructed and validated a microsimulation model to estimate changes in weight and diabetes risk from the tax, examining how changes in modeled outcomes resulted from a variety of alternative assumptions. Our a priori hypothesis was that urban populations would be the primary beneficiaries of SSB taxation, given their high SSB exposure as well as elevated obesity and type 2 diabetes prevalence rates [9],[26].

## Methods

Our analysis proceeded in three steps. First, we calculated changes to overall beverage expenditure as well as own-price and cross-price elasticities between SSBs and the other major beverage types consumed in India (milk, fresh fruit juices, coffee, and tea) using survey data relating price to consumption. We next used these elasticity estimates to calculate per capita kilocalorie and glycemic load changes expected from a 20% excise tax on SSBs. Finally, constructing a discrete-time microsimulation model, we simulated changes in overweight, obesity, and type 2 diabetes incidence and prevalence over the period 2014–2023 given changes in caloric intake and glycemic load. Each component of our analysis was stratified by age-band, sex, income, and urban/rural residence, in order to analyze disparities between demographic subpopulations in India.

### Data Sources

#### Elasticity calculations

The Indian National Sample Survey (NSS), wave 2009/2010 (the most recent data available), was used to calculate own- and cross-price elasticities corresponding to changes in SSB price in each demographic subgroup, controlling for changes in SSB availability [25]. The NSS Consumer Expenditure module is a widely used repeated quinquennial cross-sectional survey of household food consumption data from a nationally representative sample in terms of age and income distribution [25]. NSS data include beverage amount consumed and prices paid for each of the beverage categories, based on surveys of 100,855 households interviewed through a validated interviewer-assisted questionnaire with district-level validation of reported prices and oversampling of low-income, rural, and female-headed households. Our power calculations estimated that we could detect the odds ratio equivalent of a 10 kcal/person/day change with >80% power in each subgroup given a survey design effect of two [27]. We converted grams of consumption per capita into kilocalories per capita (mean and 95% confidence intervals) using a standard nutrient tables [28],[29]. Kilocalorie and glycemic load conversions included both mean and 95% confidence intervals reflecting the distribution of milk among whole, skim, and toned varieties [20]; the available fresh fruit juices on the Indian market [9]; and typical added sugar content to consumer-brewed coffee and tea [28]. Price was expressed in 2010 Indian rupees, adjusted through GDP price deflators [30].

#### BMI distribution and type 2 diabetes status

**Figure 2:**
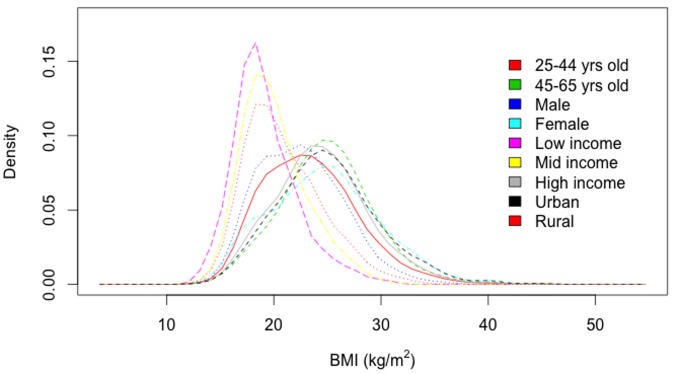
Body mass index (BMI) distributions among cohorts (kg/m^2^), 2010 [31].

While the NSS provides data on consumption and price, it does not provide data on health parameters such as BMI and type 2 diabetes status. To analyze the covariance of SSB consumption with BMI and type 2 diabetes status, we used data from the Public Health Foundation of India's Indian Migration Study (IMS) 2007–2010, a national sample of 7,049 men and women from all three income tertiles and both urban and rural residency status (Figure 2). These individuals were evaluated through interviewer-administered food frequency questionnaires and anthropometric and medical assessments published previously [20],[31]. The dietary assessment was validated against independent surveys and a subsample analysis of 418 participants subjected to three 24-hour dietary recalls [20].

### Elasticity Calculation Details

Given the absence of a suitable instrumental variable to link with SSB prices, we calculated elasticities among the beverages using the classical Quadratic Almost Ideal Demand System [32], a standardized microeconomic system of equations that estimate how price variations affect expenditure, substitution between goods, and overall consumption (the inflationary effects of taxation). The equations are detailed in Text S1, and complete elasticity results are presented for each demographic subgroup in Table 1. A standard two-step procedure was also used to first estimate the probability of consumption, to account for censoring and zero consumption, and then estimate the share of expenditures spent on each beverage, controlling for availability and a series of socioeconomic variables detailed in Text S1 [33]–[35]. The system allows us to estimate shifting out of the purchased beverage market in the context of prices (e.g., to tap water), versus the degree of substitution between beverage classes following a price change in each beverage. The demand system was estimated in Stata version MP12.1 (StataCorp). The face validity of own-price SSB elasticity was compared against an international systematic review of elasticities [36]; our demand system revealed an own-price elasticity of SSBs of −0.94 (95% CI −0.90 to −0.98), within the review-based 95% confidence interval of −0.33 to −1.24. Cross-elasticities, as tabulated in Text S1, were also similar to published estimates, although the published estimates available have not included India [37].

**Table 1:** Elasticity given 1% change in SSB price, calculated from [25].

Beverage	Income Level			Residential Sector		Overall Population (95% CI)
	Low (95% CI)	Mid (95% CI)	High (95% CI)	Urban (95% CI)	Rural (95% CI)	
*n*	28,207	50,989	21,659	59,119	41,736	100,855
Milk	0.055 (0.013–0.096)*	0.046 (0.010–0.083)*	0.046 (0.010–0.083)*	0.049 (0.010–0.087)*	0.049 (0.012–0.087)*	0.049 (0.011–0.087)*
SSBs	−0.90 (−0.86 to −0.93)*	−0.96 (−0.92 to −1.00)*	−0.96 (−0.92 to −1.00)*	−0.94 (−0.90 to −0.98)*	−0.94 (−0.90 to −0.98)*	−0.94 (−0.90 to −0.98)*
Fresh fruit juice	0.32 (0.31–0.36)*	0.30 (0.25–0.35)*	0.30 (0.25–0.35)*	0.31 (0.27–0.35)*	0.31 (0.27–0.35)*	0.31 (0.27–0.35)*
Coffee	0.0016 (−0.077 to 0.051)	0.0054 (−0.058 to 0.084)	0.0054 (−0.058 to 0.084)	0.0041 (−0.064 to −0.073)	0.0041 (−0.064 to 0.073)	0.0041 (−0.064 to 0.073)
Tea	0.10 (0.062–0.131)*	0.14 (0.12–0.18)*	0.14 (0.12–0.18)*	0.13 (0.098–0.16)*	0.13 (0.098–0.16)*	0.13 (0.098–0.16)*

*p*<0.05.

### Tax Effect Estimates

To examine the kilocalorie changes attributable to a 20% SSB tax, the price elasticity for each beverage (percent change in consumption for each 1% change in SSB price) was multiplied by the change in SSB price (20% in the baseline case) and multiplied by baseline daily kilocalorie intake among multiple Indian subpopulations to estimate the change in individual daily intake for each beverage (Table 2).

**Table 2:** Kilocalorie consumption per day from beverages, overweight and obesity prevalence (% BMI≥25 kg/m^2^), and baseline diabetes incidence (per 100,000 persons) among cohorts, used for model initialization [20],[25],[31],[41],[42].

Cohorts		Milk (SD)	SSBs (SD)	Fresh Fruit Juice (SD)	Coffee (SD)	Tea (SD)	% BMI≥25 kg/m^2^ (SD)	Diabetes Incidence (per 100,000)
Age	25–44	200 (5)	52 (1)	36 (0.3)	19 (2)	83 (5)	156 (35)	34 (8)
	45–65	226 (5)	37 (1)	30 (0.7)	24 (2)	81 (6)	379 (50)	48 (11)
Sex	M	220 (4)	50 (1)	34 (0.3)	21 (2)	85 (5)	507 (46)	32 (8)
	F	193 (5)	42 (1)	34 (0.5)	21 (2)	79 (6)	103 (44)	46 (10)
Income	Low	139 (6)	44 (1)	29 (0.8)	17 (2)	82 (6)	105 (67)	11 (2)
	Mid	173 (6)	48 (1)	40 (0.5)	26 (2)	81 (6)	157 (50)	16 (4)
	High	220 (6)	47 (1)	32 (0.7)	19 (2)	85 (6)	350 (45)	43 (10)
Residence	Urban	223 (6)	48 (1)	38 (2)	25 (2)	83 (6)	372 (50)	48 (11)
	Rural	187 (6)	45 (2)	28 (2)	16 (2)	82 (7)	242 (42)	21 (5)
Overall		207 (5)	46 (1)	34 (0.4)	21 (2)	82 (5)	38 (8)	307 (45)

Note that “income” here is measured using the Standard of Living Index (SLI), a household level asset-based scale devised for Indian surveys [38].

SD, standard deviation.

To estimate the potential effects of the tax on overweight and obesity prevalence (BMI≥25 kg/m^2^) and type 2 diabetes incidence, we constructed a microsimulation model, which simulates 10,000 adults for each cohort defined by every combination of: age (25–44, 45–65 years old), sex, income (low, middle, and high Standard of Living Index [SLI], a household-level asset-based scale devised for Indian surveys [38]), and urban/rural status (using the World Bank definition of urban residence [39]). Model details are itemized here according to ISPOR reporting guidelines [40], and the model flow diagram is depicted in Figure 2.

Sampling from the joint distribution of weight, height, consumption of each beverage type, and type 2 diabetes status from the IMS study, the model assigns simulated individuals a baseline profile of these factors, updating the estimates for secular trends (Table 2) [31],[41],[42]. Unlike a typical Markov model, the microsimulation approach can capture the impact of interventions on individual risk factor profiles, not just the average population effect of an intervention—allowing for complex relationships among multiple co-morbid risk factors to be incorporated into the experiment. This is important because reducing SSB consumption in an individual who has a high baseline intake of SSBs but also a high consumption of other beverages may have different outcomes than reducing SSB consumption for someone with less consumption of other beverages. The model was validated by comparing historical projections of 2000–2010 obesity and type 2 diabetes prevalence in India given year 2000 input values against independent World Health Organization survey-based estimates (Figure S1) [26].

We first simulated a baseline (no tax) case in which secular trends in kilocalorie consumption, glycemic load intake, and associated BMI and type 2 diabetes incidence changes were estimated. Two baseline scenarios were modeled: (1) a linear rise in SSB consumption of 13% per annum, fitting the secular trend from 1998–2012 (the longest time series available), and (2) a nonlinear rise predicted by a Bass marketing model used commonly by industry for projecting sales growth [43] (both shown in Figure 1; Bass model equation in Text S1). The model also incorporated secular trends in non-beverage calorie intake given by UN Food and Agricultural Organization estimates, to account for other caloric changes; linear trends in non-SSB beverage consumption were not statistically significant (Text S1) [9],[44]. Consumption changes were also converted into changes in glycemic load using standard glycemic index tables (Table 3) [45]. Note that these estimates include the typical distribution of sugars added by consumers to coffee and tea.

**Table 3:** Effective glycemic load (g) per kcal when accounting for typical serving sizes (g) and energy content (kcals) of beverages [45].

Beverage	Glycemic Load (g) per kcal (95% CI)
Milk	0.0311 (0.0235–0.0387)
SSBs	0.1584 (0.1408–0.1759)
Fresh fruit juice	0.0870 (0.0758–0.0981)
Coffee	0.0919 (0.0850–0.0989)
Tea	0.0553 (0.0497–0.0608)

**Figure 3:**
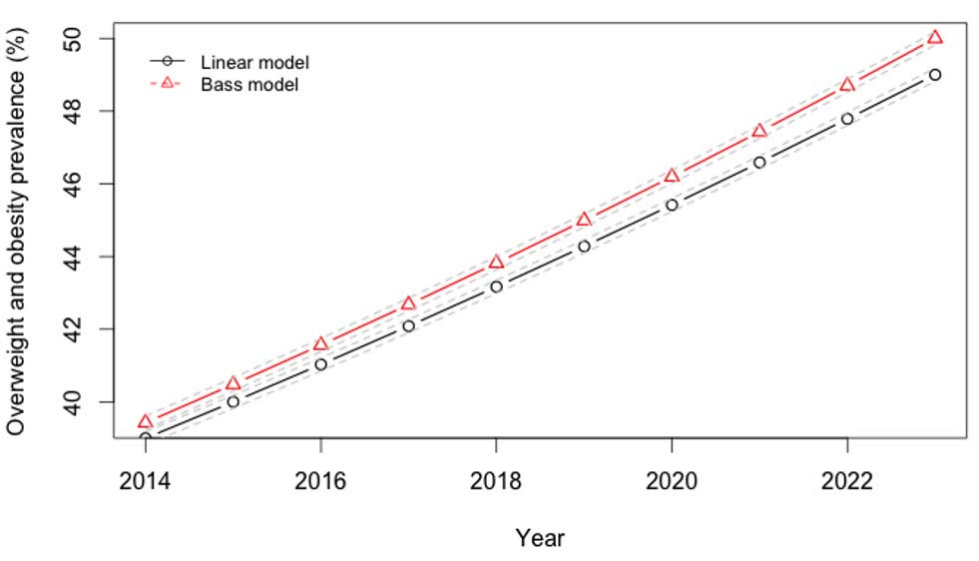
Projected trajectory of overweight, obesity in India, 2013–2024.

To convert the calorie change estimates into changes in weight over time (Figure 3), we used a validated set of equations developed by the National Institutes of Health to estimate individual body weight change after a change in calorie consumption (reproduced in Text S1 with parameter values in Table S1) [46]. While there are many potential alternative models relating caloric intake changes to body weight, we chose this model as these equations were validated against experimental controlled feeding studies among humans in the age groups included in this simulation, and more accurately predicted changes in body weight from measured changes in energy intake than did alternative published models in head-to-head comparisons [47]. The equations account for the time delay between consumption changes and weight changes, assuming that energy must be conserved, and that changes in body composition and body weight result from imbalances between the intake and utilization rates of calories along with shifts between intracellular and extracellular compartments.

**Figure 4:**
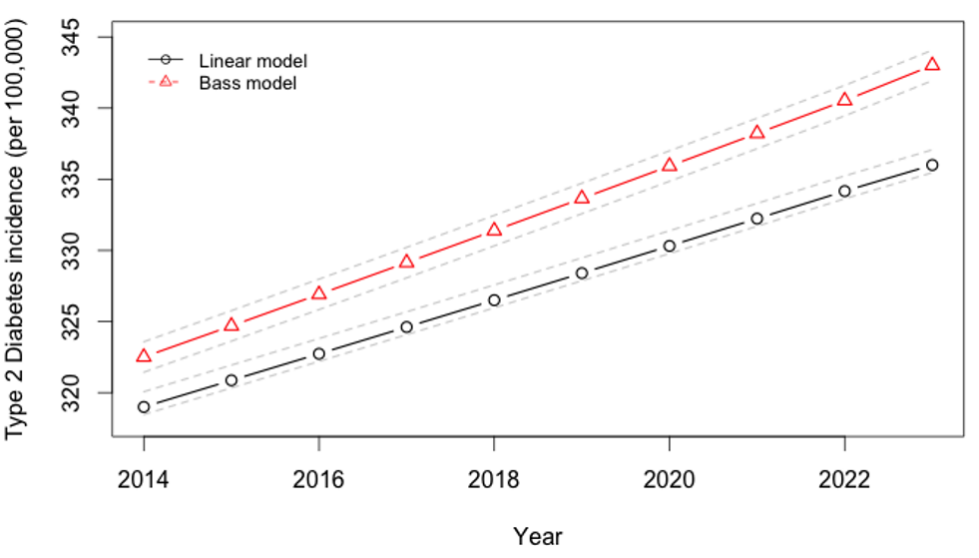
Projected trajectory of type 2 diabetes incidence in India, 2013–2024.

To estimate type 2 diabetes incidence (Figure 4), we employed a standard, validated hazard calculation method (Text S1) [48]. This calculation estimates how much an individual's risk of type 2 diabetes changes given changes in their beverage intake, employing an estimate of the relative risk of type 2 diabetes contributed by glycemic load, adjusted for an exponential rate of effect of 1/7.6 years^−1^ (95% CI 1/2.8–1/14.7 years^−1^) [49]. We used a type 2 diabetes relative risk estimate of 1.45 (95% CI 1.31–1.61) for each 100-g increment in glycemic load, based on a meta-analysis of 24 prospective cohort studies (*p*<0.001; 7.5 million person-years of follow-up) [50]. This relative risk estimate incorporates both the type 2 diabetes incidence risk associated with adiposity due to consumption, and the indirect pancreatic and hepatic effects of glycemic consumption that are obesity-independent (both obesity-mediated and non-obesity-mediated pathways) [51],[52]. We chose to use glycemic load relative risk estimates rather than relative risk estimates of diabetes specifically calculated only for SSBs [3], to account for the metabolic effects of beverages substituted for SSBs. This would be expected to produce conservative results from our simulation. Furthermore, the glycemic load calculation accounts for the fact that the impact on diabetes of different types of calories is different; that is, because the glycemic load per calorie is much higher for SSBs than other beverages (Table 3), a net change in calories alone does not predict type 2 diabetes risk, and the glycemic load estimate is used to account for the fact that some calories confer higher risk than others.

**Figure 5:**
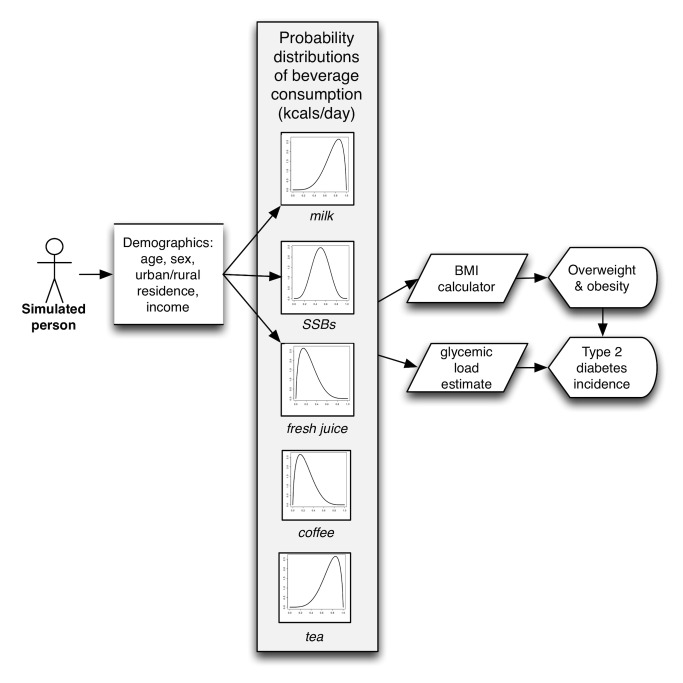
Model diagram.

For prospective simulation of the period 2014–2023, 10,000 simulations were performed of the overall model (10,000 simulations each with 10,000 individuals per cohort) in MATLAB version R2013b (MathWorks), sampling repeatedly from the probability distributions of the input parameter values to estimate 95% confidence intervals around modeled outcomes (Figure 5). All model parameters—including kilocalorie consumption, elasticities, glycemic load, relative risks, and the metabolic parameters—were included in the uncertainty analysis.

To simulate the 20% excise SSB tax, we simulated full country-wide tax coverage starting at the beginning of the year 2014. In sensitivity analyses, we varied the SSB tax rate from 10% to 30%. In a further sensitivity analysis, SSB consumption trends were simulated using a standard Bass diffusion model employed by industry to project sales growth (Figure 1, *R^2^* = 0.98) [43], rather than the baseline linear trend also shown in Figure 1.

For outcomes analysis, we computed both overweight and obesity prevalence, because the threshold of 25 kg/m^2^ has been the Indian government standard for BMI surveillance [53], given elevated risk of type 2 diabetes among South Asians at lower BMI levels (>24 kg/m^2^) [54] than the international obesity threshold of 30 kg/m^2^.

### Ethics Statement

Ethics committee approval for the IMS Study that was used to inform the model was obtained from the All India Institute of Medical Sciences Ethics Committee, reference number A-60/4/8/2004; for the overall modeling research, ethics committee approval was obtained from the Stanford University Institutional Review Board, reference number eP-28811.

## Results

### SSB Consumption, Obesity, and Type 2 Diabetes Rates

We observed little variation in SSB consumption levels among demographic cohorts in India (Table 2). Among the 390 kcal/person/day typically consumed from beverages among surveyed Indians, approximately 12% (46 kcal/person/day) were conferred by SSBs. Consumption varied from 9% (37 kcal) among the older cohort of 45–65 year olds to 13% (52 kcal) of overall beverage consumption among the younger 25–44 year old cohort, and was roughly equal among urban (12%, 48 kcal) and rural populations (12%, 45 kcal). SSBs composed 14% of beverage calories among the poorest income tertile and 12% among the wealthiest tertile. However, overall beverage calories were lowest among the poor (310 kcal/person/day) versus the wealthiest tertile (404 kcal/person/day), hence absolute consumption varied insignificantly by wealth (44 versus 47 kcal/person/day).

Rates of obesity and type 2 diabetes universally increased across cohorts in our projections over the period 2014–2023. We observed that if linear secular trends in SSB consumption continued in the absence of a tax (Figure 1), Indian overweight and obesity prevalence (percent adults 24–65 with BMI≥25 kg/m^2^) would be expected to increase from 39% to 49% and type 2 diabetes incidence would be expected to rise in parallel from 319 to 336 per 100,000 per year over the period 2014–2023. Amplification of these trends to 50% overweight prevalence and 343 per 100,000 type 2 diabetes incidence by year 2023 were observed in the Bass diffusion scenario, in which SSB consumption followed the curvilinear rise of marketing model projections (Figure 1), which forecast consumption increasing from 12.8 l/person/year in 2014 to 36.3 l/person/year in 2023 (approximately one-fourth the 2012 rates in Latin American countries [9]; Figure 1).

### Elasticities

Much of the rise in SSB consumption would be expected to shift toward other beverage consumption in the context of an SSB tax. On the basis of microeconomic demand system estimates of expenditure data, SSB consumption was observed to decline by 0.94% for each 1% increase in SSB price (95% CI, a 0.90%–0.98% reduction). Substitution among beverages revealed a 0.049% (0.011%–0.087%) increase in milk consumption, 0.31% (0.27%–0.35%) increase in fresh fruit juice consumption, and a 0.13% (0.098%–0.16%) increase in tea consumption for each 1% rise in SSB price in the overall population, with small variations between groups (Table 1), but a non-significant degree of substitution with coffee (0.004%, −0.064% to 0.073%). Full elasticity estimate details are provided in Table 1.

### Tax Effects

**Figure 6:**
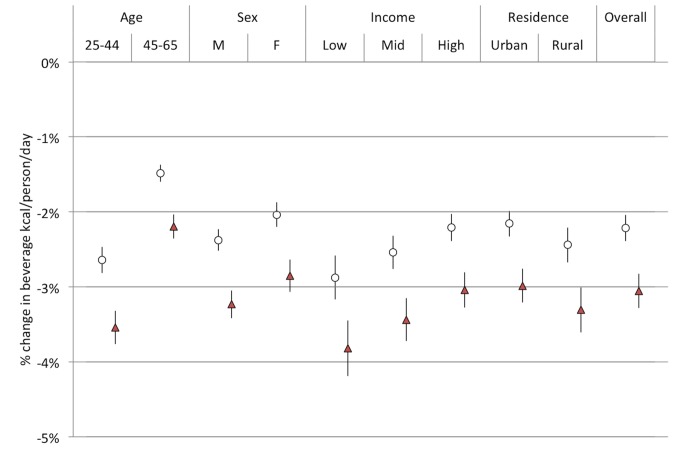
Projected changes in kilocalories per person per day consumed from all beverages. Open circles, linear model; red triangles, Bass model of changes in SSB consumption over time.

**Figure 7:**
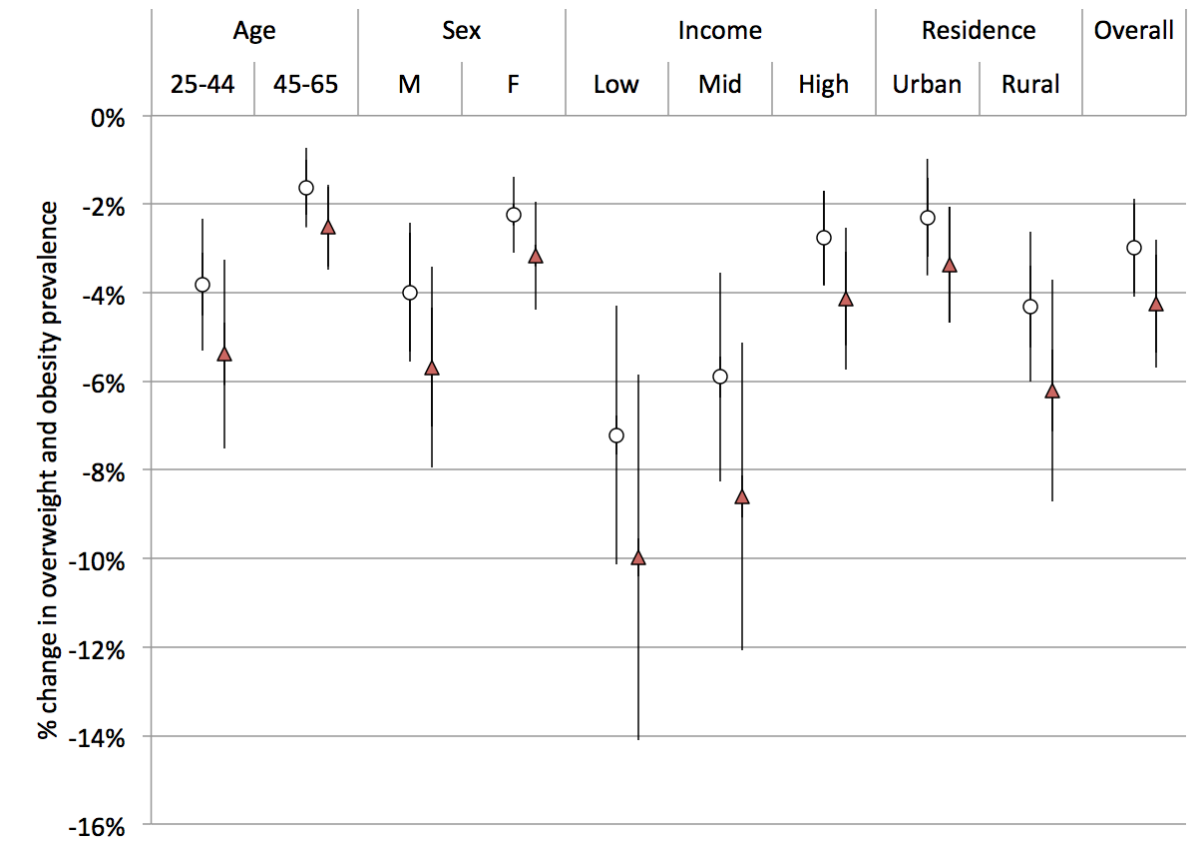
Change in overweight and obesity prevalence (relative change in percent of adults with body mass index >25 kg/m^2^). Open circles, linear model; red triangles, Bass model of changes in SSB consumption over time.

**Figure 8:**
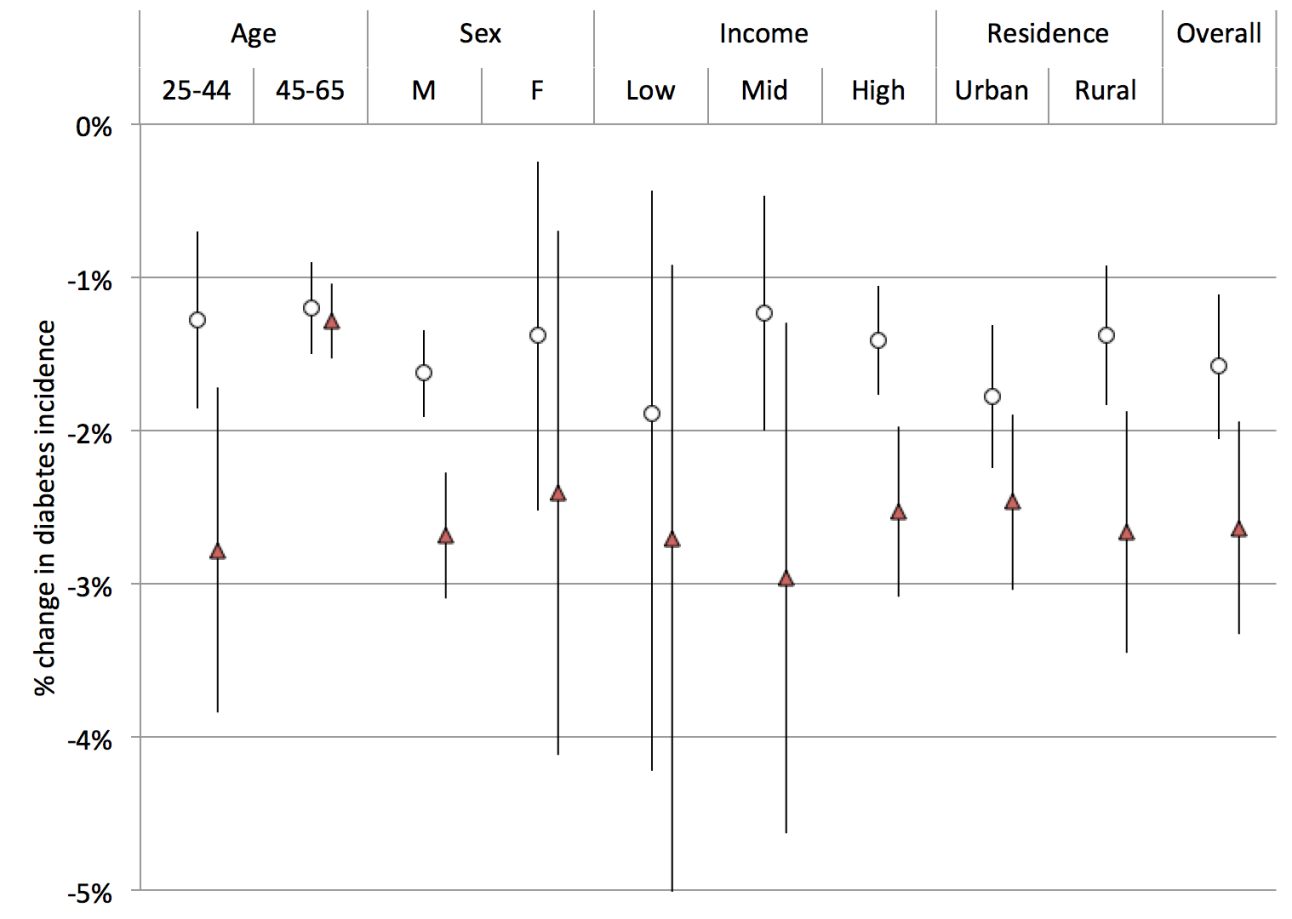
Change in type 2 diabetes incidence (per 100,000) under a 20% excise tax on SSBs. Open circles, linear model; red triangles, Bass model of changes in SSB consumption over time.

Using the calculated elasticities to project the effects of a 20% excise tax on SSBs (Figures S2–S6), we estimated obesity and type 2 diabetes rate changes among demographic cohorts (Figures 6–8). Overweight and obesity prevalence declined by 1.6% to 5.9% and type 2 diabetes incidence by 1.2% to 1.9% from the baseline estimates among the Indian subpopulations under a 20% SSB tax (Figures 7 and 8; 3.0% overweight/obesity reduction and 1.6% type 2 diabetes reduction in the overall population). Different sensitivities to the tax among cohorts were driven primarily by differences in the distribution of BMI, such that groups with lower current median BMIs were more easily able to maintain members of the cohort below the threshold of 25 kg/m^2^ (Table 2). In the setting of linear consumption increases in SSBs, younger (25–44 year olds), male, low-income, and rural populations were observed to experience the largest relative decline in kilocalorie consumption from beverages and associated declines in overweight and obesity prevalence (Figures 6–8). When differential glycemic load among beverages and different baseline incidence rates of type 2 diabetes were accounted for, urban rather than rural populations experienced the largest relative declines in type 2 diabetes incidence (Figure 8). As shown in Figures 6, 7, and 8, a large confidence interval was observed among females and low-income populations due to imprecision in current diabetes incidence estimates among these groups, resulting from less robust surveillance quality among these cohorts.

Converting the relative rate declines into absolute numbers of averted overweight, obesity, and type 2 diabetes cases—accounting for population size differences and demographic trends in population growth [55]—revealed large variations among population subgroups. The largest number of prevalent overweight and obesity cases averted from 2014–2023 would still be expected among the younger cohort (3.9 million people avoiding overweight in the 25–44 year group versus 1.1 million in the 45–65 year old group), as well as males (2.9 million versus 2.1 female), and rural populations (3.1 million versus 1.4 million urban), but also among the highest income tertile (1.7 million versus 1.4 million in the mid tertile and 1.1 million among the lowest tertile). In absolute numbers, the most type 2 diabetes cases averted from 2014–2023 were among the older cohort (573,000 among 45–65 year olds versus 477,000 in 25–44 year olds over 2014–2023), men (1.6 million versus 1.2 million women), the highest income tertile (603,000 versus 248,000 in lowest tertile), and rural populations (877,000 versus 741,000 urban). In total, 11.2 million overweight and obesity cases (−3.0%, 95% CI 7.5–15.0 million) and 400,000 type 2 diabetes cases (−1.6%, 95% CI 300,000–500,000) would be averted from 2014–2023 by a 20% SSB excise tax, according to our model.

### Sensitivity Analyses

The preventive impact of the tax was amplified by 40% to 60% when we shifted our assumptions from a linear trend in SSB consumption to a Bass diffusion model of SSB consumption trends, but qualitative differences between population subgroups were unaltered (Figures 6–8). Should consumer trends in SSB consumption increase according to the Bass trajectory, the preventive efficacy of a 20% SSB tax would rise by 40% to 60% over the baseline forecast, reducing overweight and obesity prevalence by 2.5%–10.0% and averting 1.0%–2.8% of incident type 2 diabetes (a 4.2% reduction in overweight/obesity prevalence, or 15.8 million people, 95% CI 10.4–21.1 million, and 2.5% reduction in type 2 diabetes incidence in the overall population, or 600,000 people, 95% CI 400,000–800,000; Figures 7 and 8).

The sensitivity of overweight, obesity, and type 2 diabetes to tax rate variations was linear in the case of overweight and obesity rates, but nonlinear for type 2 diabetes incidence, such that reducing the tax from 20% to 10% reduced the projected impact of the tax on overweight and obesity by 50% and on type 2 diabetes incidence by 62%. Conversely, increasing the tax from 20% to 30% increased the projected impact of the tax on overweight and obesity by 50% and on type 2 diabetes incidence by 33%. The nonlinearity in diabetes rates arose from complex changes in glycemic load intake when accounting for substitutions between beverages; increasing the SSB tax, for example, produces diminishing returns as substituted goods contribute to total glycemic load consumption to varying degrees, conditional on their price and total per capita expenditures on beverages.

## Discussion

An excise tax on SSBs would be expected to mitigate increases in overweight, obesity, and type 2 diabetes cases in India under numerous alternative scenarios and assumptions, even after accounting for beverage substitution patterns. Under the conservative scenario of a linear rise in SSB consumption, a 20% SSB excise tax would be expected to prevent 11.2 million new cases of overweight and obesity (a 3.0% decline), and 400,000 cases of type 2 diabetes (a 1.6% decline) over the decade 2014–2023, according to a microsimulation model informed by nationally representative consumer expenditure, price, BMI, and type 2 diabetes incidence data. Under the case in which consumption follows business marketing models, which mirror recent SSB sales increases, a 20% SSB tax would be expected to avert 4.2% of prevalent overweight/obesity and 2.5% of incident type 2 diabetes from 2014–2023. As compared to other population-level obesity interventions (e.g., nutrition labels and consumer education) that have been studied to date, which typically result in <1% reductions in overweight and obesity and non-significant changes in diabetes rates [56]–[60], this implies a comparatively large population-level impact from SSB taxation. While a number of low- and middle-income countries are creating an array of large-scale interventions to address increased obesity and diabetes, to date none have sustained reductions in BMI [18].

The SSB tax appeared likely to significantly lower both BMI and type 2 diabetes incidence among all demographic cohorts, without perverse increases in BMI or type 2 diabetes due to substitution effects. However, the effect sizes of the tax varied notably among different demographic groups. Contrary to our a priori hypothesis, the largest relative declines in overweight and obesity prevalence were observed among young rural men, as this group with lower current BMI more easily maintained itself below the BMI threshold of 25 kg/m^2^ in scenarios with SSB taxation than without such taxation. The BMI threshold of 25 kg/m^2^ corresponds to a critical inflexion point of increasing type 2 diabetes risk among Indians [54].

These findings offer substantial contributions to the existing literature on non-communicable disease prevention in low- and middle-income countries. Our assessment is the first to our knowledge to study the impact of SSB taxation in India, which is expected to experience more deaths from non-communicable diseases than any other country in the world over the next decade, and is considered a policy leader among developing nations devising chronic disease interventions [61]. Unlike most assessments of large-scale interventions in developing countries, our study is based on disaggregated population-representative data specific to different income groups, urban/rural residence, both sexes, and both middle-aged and older adults, accounting for within-country heterogeneity in consumption behavior and disease risk. Prior policy models have been criticized for either projecting results from Western populations onto other countries, or aggregating large, heterogeneous developing country populations into a single population average, which can produce perverse outcomes when policies benefit one segment of the population while potentially risking poor outcomes among others [17],[62].

Our results also incorporate the effects of substitution among beverage classes through a direct estimation method rather than assumptions alone, an advance over most models of fiscal policy that have been criticized for ignoring this issue or assuming arbitrary levels of substitution [63]. The research also lends insights into the fact that obesity and diabetes impacts of SSB taxes may not be entirely parallel, in light of the glycemic load effects of food intake on type 2 diabetes risk. This glycemic load factor incorporates the differential impact of each calorie of SSBs versus other beverages on diabetes risk, given that glycemic load per calorie of SSBs is about 5.3 times that of milk, for example (Table 3).

The impact of BMI on chronic disease among Indians is notably different than among other populations. A large literature suggests that Indians at BMIs ranging from 20–22 kg/m^2^ have percent body fats equivalent to non-Hispanic white Americans or British adults with BMIs of 27–30 kg/m^2^, and Indians have an increased risk type 2 diabetes at much lower BMI levels than these other populations [64]–[68]. Our estimates therefore relied directly on both adiposity-related and direct metabolic impact estimates of type 2 diabetes risk associated with glycemic load consumption changes. The estimates incorporate the glycemic load impact of fruit juices as substitutes for SSBs, given the literature suggesting that fruit juice consumption may have adverse metabolic effects consistent with glycemic load contributions, even if having lower calories than SSBs [69].

As with other projections of fiscal policy interventions, our assessment relies on mathematical modeling, which inherently requires several assumptions and limitations. First, we employ the assumption that consumer expenditure behavior from prior years, captured in price elasticities, will reflect future behavior among consumers. This abstraction makes it impossible to account for the potential increased willingness-to-pay for SSBs in the context of social trends in popularity and income increases. Second, our metabolic equations calculating weight change in the context of caloric change does not account for diet beverages (which are currently <0.1 l/person/year in India [9]) that have unclear relationships to metabolic syndrome [70]–[72], and assumes that physical activity will not change directly as a result of soda taxation, even though compensatory activity after substitution may also occur (e.g., individuals who change their diet may decide to exercise more or less based on perceptions of the healthfulness of their dietary change). Third, we abstract from dietary food frequency questionnaires that are validated against 24-hour dietary recalls and independent databases [20], but are still subject to recall bias and underreporting. Fourth, our model produced wide confidence intervals among the lowest-income tertile and women due to undersampling of rural low-income populations. Nevertheless, our purpose in employing this model was not to predict exact future rates of disease, which is impossible from any model, but to understand potential demographic differences in taxation impact and estimate the sensitivity of forecasts to varying assumptions about future SSB consumption. A consistent finding among all cohorts was that a rise in SSB consumption in accordance with recent trends would portend increasing overweight, obesity, and type 2 diabetes rates, but also render an excise tax on SSBs differentially more effective as a preventive population strategy. Finally, we did not account for safety concerns if SSB taxation shifts to increasing tap water usage in the context of some populations have unsafe water supplies in India; however, this is unlikely to produce a true epidemiological shift in disease burden as populations already exposed to non-potable water-based pathogens would likely to continue to be exposed, and unexposed populations are unlikely to be newly exposed because of an SSB tax given that nearly all populations drink some tap water in their locality. Similarly, we did not track the vitamin and mineral-related implications of SSB taxation as it implies differential consumption of fruit juices that may have other nutritional benefits but that also contribute to type 2 diabetes risks [69].

Another limitation of our analysis is that our treatment of the SSB taxation strategy is unable to quantify the attendant ethical, political, and social dilemmas presented by taxation strategies. Sufficient data are not available on changes in beverage intake behaviors among Indian children, or the long-term metabolic and cardiovascular consequences of SSB consumption changes among children aside from weight gain [73]. Hence, we focused on validated models of adult metabolism, since the cardiovascular and metabolic disease burden and health care cost would be expected to accrue most among adults over the near-term policy window that we simulated here. Excise taxation on foods can also be viewed as discriminatory, paternal, or regressive (in an economic sense). An alternate perspective is that preventing obesity and diabetes among lowest-income populations, who are among the most affected over time, will produce the greatest social benefit as low-income populations are also least likely to obtain diagnosis and treatment for chronic disease [5]. Another unresolved political issue is the administrative challenge of enforcing taxes on purchases in informal settings, given that SSBs are often sold by small vendors, with potential implications for household income, economic growth, and poverty given the employment impact of SSB sales. Excise taxes at the manufacturing level would allow bypassing of some enforcement obstacles, but remain politically opposed by beverage companies. Studies of existing SSB taxes in Western populations have highlighted that the taxes imposed have been generally too small to have meaningful effect size, while imposing larger taxes at equivalent levels to those simulated here may confer greater benefits [14],[15]. Future research should replicate the findings observed here in other rapidly developing middle-income countries where SSB consumption is increasing at a rapid rate [6].

For policy, our research indicates that SSB price increases are likely to generate substantial reductions in overweight, obesity, and type 2 diabetes through pathways affecting caloric intake and glycemic load. Fiscal strategies could mitigate obesity and type 2 diabetes in India over the next decade, even for more remote and low-income populations that are less likely to have transitioned to other components of Western diets in the near term.

## Supporting Information

Figure S1 **Model-based estimates of prevalence of overweight (BMI>25 kg/m^2^) and type 2 diabetes prevalence versus WHO estimates** [**26**] **.**
(TIF)

Figure S2 **Model-based estimates of the probability distributions of change in milk intake after a 20% SSB tax in the baseline scenario (noting no significant change in consumption of coffee).** Consumption estimates are in units of kcals/person/day.
(TIF)

Figure S3 **Model-based estimates of the probability distributions of change in SSB intake after a 20% SSB tax in the baseline scenario (noting no significant change in consumption of coffee).** Consumption estimates are in units of kcals/person/day.
(TIF)

Figure S4 **Model-based estimates of the probability distributions of change in fresh fruit juice intake after a 20% SSB tax in the baseline scenario (noting no significant change in consumption of coffee).** Consumption estimates are in units of kcals/person/day.
(TIF)

Figure S5 **Model-based estimates of the probability distributions of change in tea intake after a 20% SSB tax in the baseline scenario (noting no significant change in consumption of coffee).** Consumption estimates are in units of kcals/person/day.
(TIF)

Figure S6 **Joint distributions of consumption change among the individual beverage classes in the baseline scenario.** Consumption estimates are in units of kcals/person/day.
(TIF)

Table S1 **Energy metabolism parameter values used in the model.**
(DOCX)

Text S1 **Modeling details.**
(DOCX)

## Abbreviations

BMI: body mass index
SSB: sugar-sweetened beverage
