# Supplementary figures and images for: Averting Obesity and Type 2 Diabetes in India through Sugar-Sweetened Beverage Taxation: An Economic-Epidemiologic Modeling Study
Source: PLoS Med. 2014 Jan 7;11(1):e1001582. doi: 10.1371/journal.pmed.1001582 (PMC3883641; doi:10.1371/journal.pmed.1001582)

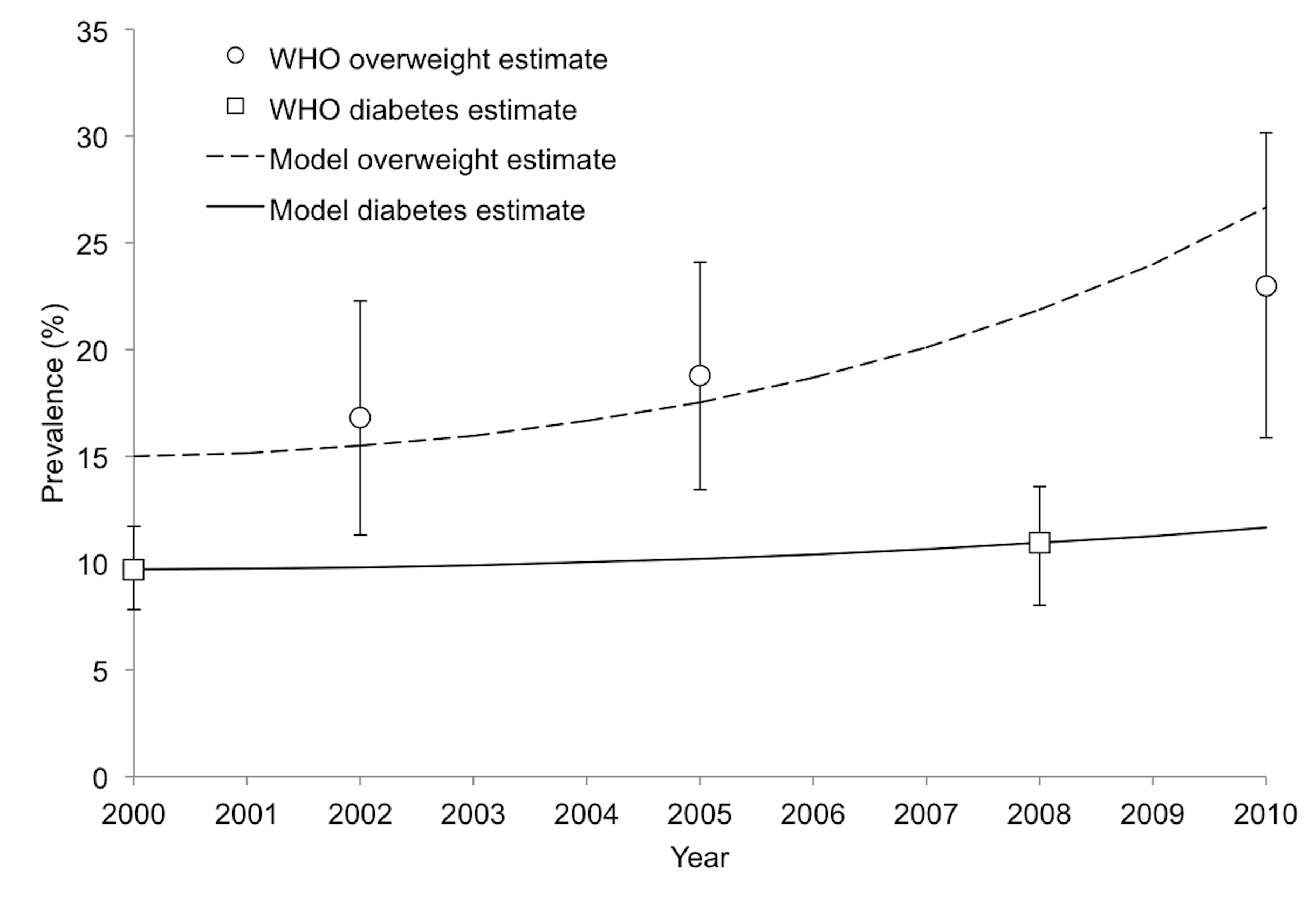

Supplement: Figure S1 — Model-based estimates of prevalence of overweight (BMI>25 kg/m2) and type 2 diabetes prevalence versus WHO estimates [26] . (TIF) [file pmed.1001582.s001.tif]

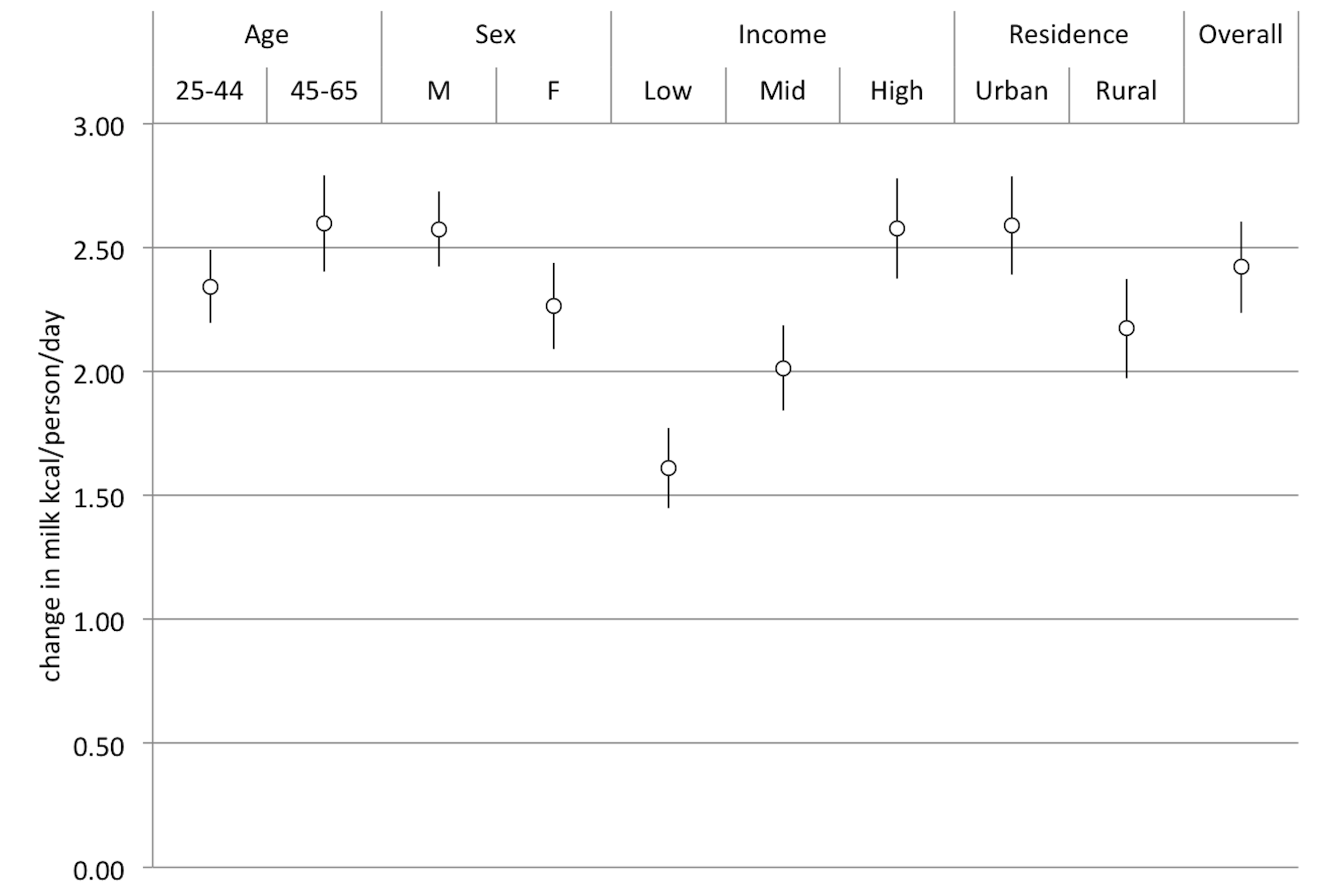

Supplement: Figure S2 — Model-based estimates of the probability distributions of change in milk intake after a 20% SSB tax in the baseline scenario (noting no significant change in consumption of coffee). Consumption estimates are in units of kcals/person/day. (TIF) [file pmed.1001582.s002.tif]

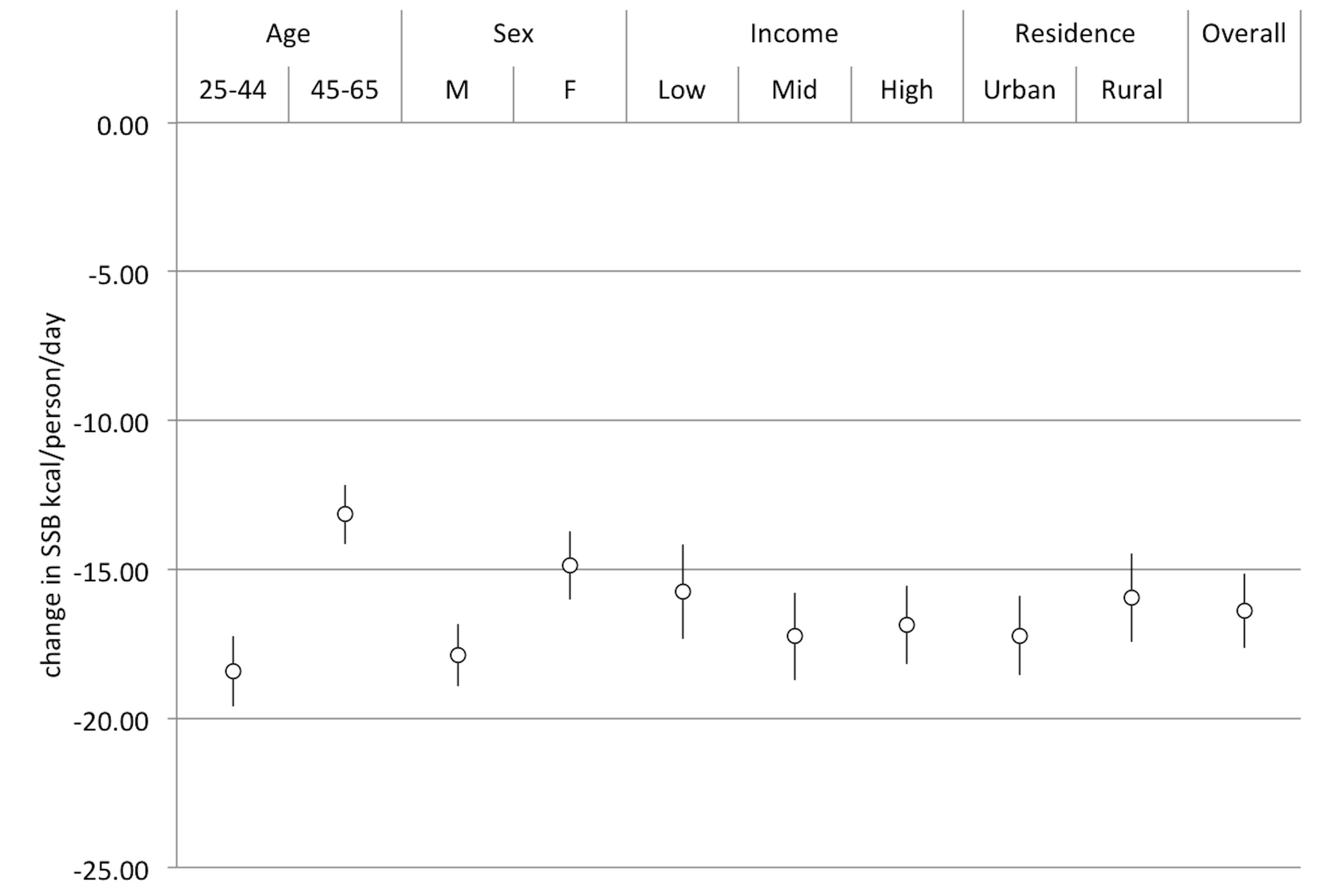

Supplement: Figure S3 — Model-based estimates of the probability distributions of change in SSB intake after a 20% SSB tax in the baseline scenario (noting no significant change in consumption of coffee). Consumption estimates are in units of kcals/person/day. (TIF) [file pmed.1001582.s003.tif]

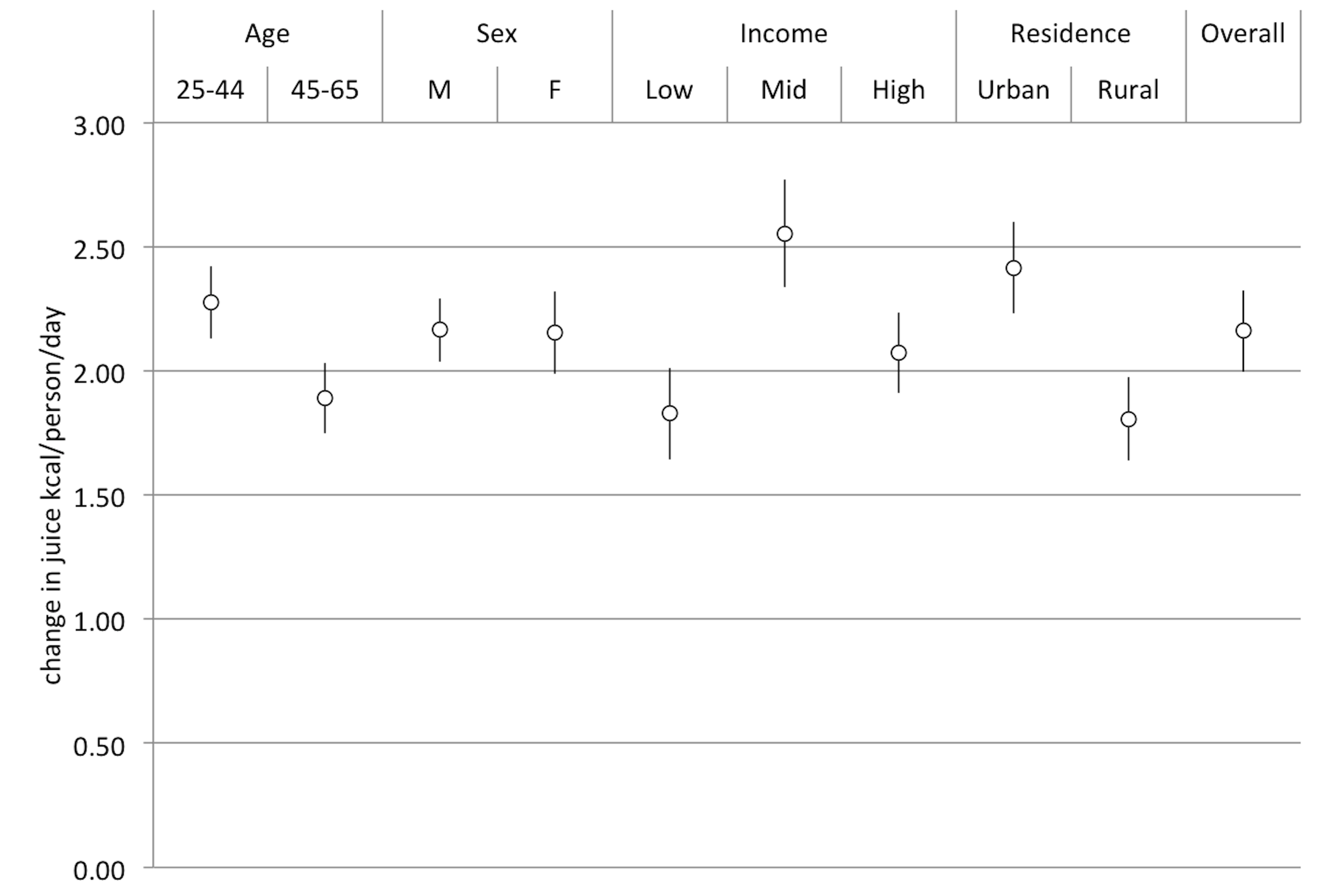

Supplement: Figure S4 — Model-based estimates of the probability distributions of change in fresh fruit juice intake after a 20% SSB tax in the baseline scenario (noting no significant change in consumption of coffee). Consumption estimates are in units of kcals/person/day. (TIF) [file pmed.1001582.s004.tif]

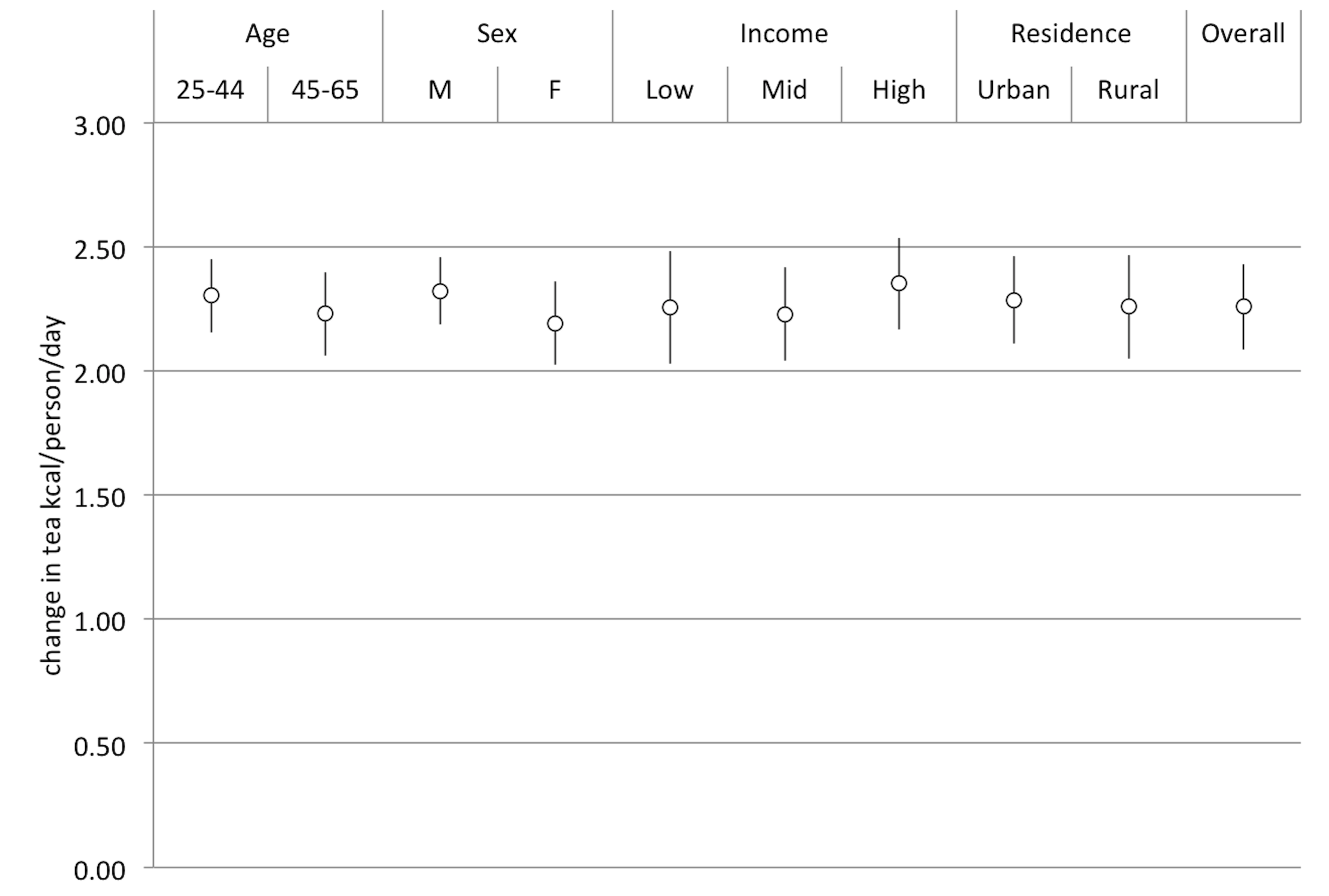

Supplement: Figure S5 — Model-based estimates of the probability distributions of change in tea intake after a 20% SSB tax in the baseline scenario (noting no significant change in consumption of coffee). Consumption estimates are in units of kcals/person/day. (TIF) [file pmed.1001582.s005.tif]

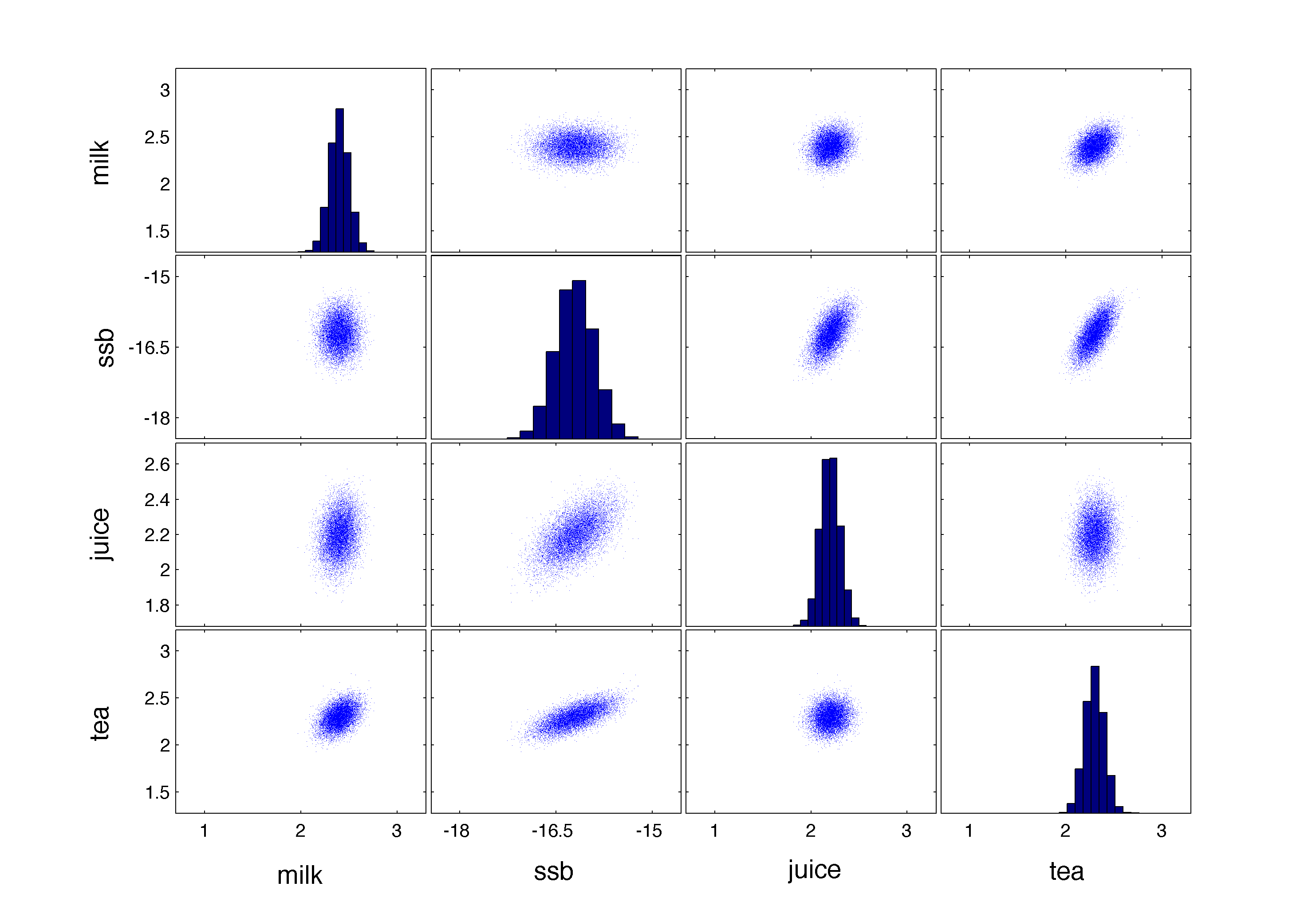

Supplement: Figure S6 — Joint distributions of consumption change among the individual beverage classes in the baseline scenario. Consumption estimates are in units of kcals/person/day. (TIF) [file pmed.1001582.s006.tif]
