# Supplementary material for: Averting Obesity and Type 2 Diabetes in India through Sugar-Sweetened Beverage Taxation: An Economic-Epidemiologic Modeling Study
Source: PLoS Med. 2014 Jan 7;11(1):e1001582. doi: 10.1371/journal.pmed.1001582 (PMC3883641; doi:10.1371/journal.pmed.1001582)
Supplement: Table S1 — Energy metabolism parameter values used in the model. (DOCX) [file pmed.1001582.s007.docx]

# Table S1. Energy metabolism parameter values used in the model, from [9,21].

| *Parameter* | *Definition* | *Value (mean+/-SD)* |
| --- | --- | --- |
| *η_f_* | Fat synthesis efficiency | 230 kcal/kg +/- 100 |
| *η_l_* | Protein synthesis efficiency | 180 kcal/kg +/- 20 |
| *ρ_f_* | Energy content per unit change in body fat | 9400 kcal/kg +/- 50 |
| *ρ_l_* | Energy content per unit change in lean tissue | 1800 kcal/kg +/- 50 |
| *c* | Relative change in lean mass per change in fat mass | 0.54 +/- 0.1 |
| *d* | Adaptive thermogenesis parameter | 0.24 +/- 0.1 |
| *γ_f_* | Resting metabolic rate of fat | 3.6 kcal/kg/d +/- 2 |
| *γ_l_* | Resting metabolic rate of lean tissue | 22 kcal/kg/d +/- 4 |
