# Supplementary material for: Averting Obesity and Type 2 Diabetes in India through Sugar-Sweetened Beverage Taxation: An Economic-Epidemiologic Modeling Study
Source: PLoS Med. 2014 Jan 7;11(1):e1001582. doi: 10.1371/journal.pmed.1001582 (PMC3883641; doi:10.1371/journal.pmed.1001582)
Supplement: Text S1 — Modeling details. (DOCX) [file pmed.1001582.s008.docx]

SI Text S1.

*Demand system equations*

The demand system specifies that the share of expenditures for a given good *i* in an *n*-good system is:

(1) for *i*=1,…,k,

where *ωi* is the share of expenditure associated with the *i*th good, *αi* is is the constant coefficient to be estimated for the *i*th share equation, *γi* and *κi* are slope coefficients to be estimated for the *j*th good in the *i*th share equation, *pj* is the price on the *j*th good, *βi* is a parameter to be estimated that will enter into the elasticity estimate, *x* is the total expenditure on beverages, *τi* is the error term, and *a, b,* and *λ* are transformations of price specified below. *Ф* and *φ* are the univariate standard normal cumulative distribution function and the probability density function, respectively, estimated from equation 2 below, which is a standard probit regression to estimate the probability that a household will consume good *i* (to account for censoring and zero consumption):

(2) $d_{ih}=\theta_{0}+\sum_{j} \theta_{ij}\ln p_{j}+\theta_{x}\ln x_{h}+\sum_{k} \theta_{nk}n_{kh}+\mu_{i}$,

where *dih* =1 if the *h*thperson consumes the *i*th good and zero otherwise, and *n* are demographic control variables (mean per-capita total expenditure as a marker of wealth, season in which the household reported consumption, household size, district of residence, household type that corresponds to major source of income, caste, urban/rural location, and land ownership status) [1]. The corresponding *Ф* and *φ* are computed as instruments for the second-stage estimation of equation 1 [2]. The price transformations include the transcendental logarithm function:

(3) $\ln a(p)=\alpha_{0}+\sum_{k=1}^{n} \alpha_{k}\ln p_{k}+\frac{1}{2}\sum_{k=1}^{n} \sum_{j=1}^{n} \gamma_{ij}\ln p_{k}p_{j}$

and a Cobb-Douglas price aggregator:

(4) $b(p)=\prod_{k=1}^{n} p_{k=1}^{\beta_{k}}$

and

(5) $\lambda(p)=\sum_{i=1}^{k} \lambda_{i}\ln p_{i}$

The demand system is constrained by the following restrictions on the parameters to ensure the parameters properly add up and follow homogeneity and Slutsky symmetry impositions:

(6) $\sum_{i=1}^{k} \alpha_{i}=1$,

(7) $\sum_{i=1}^{k} \beta_{i}=0$,

(8) $\sum_{i=1}^{k} \lambda_{i}=0$

(9) $\sum_{i=1}^{k} \gamma_{ij}=0$ for all *i* (homogeneity), and

(10) $\gamma_{ij}=\gamma_{ji}$ for all *i* and *j* (symmetry).

From this demand system, uncompensated price elasticities are estimated as:

(11) $\varepsilon_{i,j}=\frac{1}{\omega_{i}}\left\{\gamma_{ij}-\left[\beta_{i}+\frac{2\lambda_{i}}{b(p)}(\ln x-\ln a(p))\right]\left(\alpha_{j}+\sum_{k=1}^{n} \gamma_{kj}\ln p_{k}\right)-\frac{\beta_{i}\lambda_{i}(\ln x-\ln a(p))^{2}}{b(p)}\right\}-\delta_{ij}$,

where *δij* is the Kronecker delta function equal to 1 if *i*=*j* (own-price elasticity) and equals zero (cross-elasticity) otherwise. The demand system was estimated in Stata version MP12.1 (StataCorp, College Station, Texas), and the resulting parameters are listed in main text Table 1. For face validity, the elasticity estimates were compared to an international systematic review of elasticities [3], in which the own-price elasticity of SSBs varied from -0.13 to -3.18 with a 95% confidence interval upon synthesis of all 14 studies to -0.33 to -1.24 (unweighted mean -0.79 with no weighting or bias assessment in the analysis), consistent with our estimate of -0.94 (95% CI: -0.90, -0.98).

*Tax effects*

The price elasticities were used to estimate beverage consumption changes corresponding to a 20% SSB tax for comparability against tax simulations in Western populations, where a penny-per-ounce tax amounts to an ~20-25% price increase [4]; the 20% change is within the 35% SSB price variation range in the National Sample Survey (within-sample variation). While the NSS provides data on consumption and price, it does not provide data on health parameters such as BMI and type 2 diabetes status. For our model, we wished to capture the covariance (joint distribution) between consumption, BMI and type 2 diabetes since the impact of an SSB tax on population-level obesity and diabetes outcomes may be different among cohorts who have many people near the threshold for obesity rather than cohorts who are universally more lean, for example.

To capture these co-dependencies, we used data from the Public Health Foundation of India’s IMS Study, a national sample of 7,049 men and women from all three income tertiles and both urban and rural residency status, who were evaluated through interviewer-administered food frequency questionnaires and anthropometric and medical assessments as published previously [5,6]. The dietary assessment was validated against independent surveys and a subsample analysis of 418 participants subjected to three 24-hour dietary recalls [5]. To estimate the kilocalorie changes resulting from the SSB tax, price elasticity for each beverage (percent change in consumption for each 1% change in SSB price) was multiplied by the change in SSB price (20% in the baseline case) and multiplied by baseline daily intake from the IMS to get the change in individual daily intake for each beverage. Consumption estimates, displayed in Table 2 of the main text, were stratified by age-band (25-44, 45-65 years old), sex, income (low, middle and high Standard of Living Index (SLI), a household-level asset-based scale devised for Indian surveys, [7]), and urban/rural status (using the World Bank definition of urban residence, [8]).

*Microsimulation of overweight, obesity and type 2 diabetes outcomes*

To convert the calorie change estimates into changes in weight over time, we used the validated National Institutes of Health model of individual body weight *M(t)* change after a change in calorie consumption *χ*:

(12) $\frac{dM(t)}{dt}=[\chi(t)-\kappa(t)(M(t)-M_{0})]/\tau$.

where *M0*is the initial body weight prior to the calorie consumption change, *τ* is the weight change associated with net energy consumption, and *κ* captures energy expenditure [9]. The internal physiology of metabolism is captured by:

(13) $\tau=\frac{\eta_{f}+\rho_{f}+c\eta_{l}+c\rho_{l}}{(1-d)(1+c)}$ and

(14) $\kappa(t)=\frac{1}{(1-d)}\left(\frac{\gamma_{f}+c\gamma_{l}}{(1+c)}+P(t)\right)$

where Equation 13 captures the efficiency of fat and protein synthesis *nf*  and *nt*, energy content per unit fat and lean tissue *ρf* and *ρl* , relative change in lean mass per change in fat mass *c*, and adaptive thermogenesis *d.* Equation 14 describes catabolic energy breakdown given resting metabolic rates of fat and lean tissue *γf*  and *γl* and physical activity *P.* Parameter values and ranges are listed in SI Table S1. Note that we included baseline secular trends in non-beverage calorie intake per capita from UN FAO estimates to account for the non-beverage trends in increasing calorie consumption in India [10]. We assumed no change in physical activity resulting from the SSB tax.

To estimate type 2 diabetes incidence, we employed a standard hazard calculation method devised by the Institute for Health Metrics and Evaluation [11]. A simulated non-diabetic individual *j*’s relative hazard ** the hazard of becoming a type 2 diabetic in a given year in relation to the typical hazard in the cohort that year, is defined by $\delta_{j}=e^{\sum_{i} rx_{i}}$, where *r* is the log relative risk of type 2 diabetes contributed by glycemic load, adjusted for exponential rate of effect between consumption and risk of 1/7.6 years-1 (95% CI: 1/2.8-1/14.7), and *x* is load from beverage *i* for the individual. We used a relative risk estimate of 1.45 (95% CI: 1.31, 1.61) for a 100-g increment in glycemic load based on a meta-analysis of 24 prospective cohort studies (*p*< 0.001; 7.5 million person-years of follow-up) [12]. This relative risk value incorporates both the diabetes incidence risk associated with adiposity due to consumption, and the indirect pancreatic and hepatic effects of glycemic consumption that are obesity-independent (both the obesity-mediated and non-obesity-mediated pathways toward type 2 diabetes) [13,14]. We chose to use glycemic load relative risk estimates rather than relative risk estimates of diabetes specifically calculated only for SSBs [15], to account for the metabolic effects of sugars and other calories in beverages substituted for SSBs. This may lower our estimate of effect size from taxation as compared to other models.

The individual’s absolute risk *σ* in a given year is the product of their cohort-specific type 2 diabetes incidence rate *y* updated for secular trends [16] (estimated as a 0.23% change per year among 25-44 year old males, a 0.20% change per year among 45-65 year old males, a 0.68% change per year among 25-44 year old females, and a 0.25% change per year among 45-65 year old females ), multiplied by the ratio of individual relative hazard *δ* to baseline mean relative hazard * *among non-diabetics in their cohort that year: *σj* = *yj(δj/j­)*. Individual incidence was simulated probabilistically by assigning an individual new type 2 diabetes status if a random number sampled from U(0,1) was less than *σj*. The model was validated by comparing historical projections of 2000-2010 obesity and type 2 diabetes prevalence in India given year 2000 input values against independent WHO estimates (SI Figure S1) [17]. For prospective simulation of the period 2014-2023, 10,000 simulations were performed in MATLAB version R2013b (Cambridge, The MathWorks, Inc.), sampling repeatedly from the probability distributions of the input parameter values to estimate 95% confidence intervals around modeled outcomes.

To simulate the 20% excise SSB tax, we simulated full countrywide tax coverage starting at the beginning of the year 2014. The calorie intake and glycemic load estimates for each individual were changed based on the elasticity estimates as described above, to compute new trajectories for overweight, obesity and type 2 diabetes as compared to the baseline trajectory (adjusting the diabetes incidence rate per average glycemic load change). In sensitivity analyses, we varied the SSB tax rate from 10% to 30%. In the baseline simulation, we also assumed the annual consumption trend for each beverage would continue linearly from the rate observed in international sales data from 1998-2012 [18], which for SSBs was a 13.3% increased consumption per year (main text Figure 1, *R2*=0.87), but was non-significant at the p<0.05 level for other beverages. In a further sensitivity analysis, SSB consumption trends were simulated using a standard Bass diffusion model employed by industry to project sales growth [19]:

(7) $\frac{dN\left( t \right)}{dt}=\frac{q}{m}N\left( t \right)[m-N\left( t \right)]$

where *N(t)* is kilocalorie consumption per capita, *m* is the theoretical market saturation parameter, and *q* is a social diffusion parameter (note that we omit an additional marketing parameter as the extra parameter was unnecessary to fit the data in main text Figure 1, using Akaike’s Information Criterion [20], which favored the simpler model above). The estimated coefficients are *q*=0.1159 and *m*=1.011x106 based on least-squares fits to the SSB consumption time trend (Figure 1, *R2*=0.98).

#

# References

1. Pollak RA, Wales TJ (1978) Estimation of complete demand systems from household budget data: the linear and quadratic expenditure systems. Am Econ Rev 68: 348–359.

2. Shonkwiler JS, Yen ST (1999) Two-step estimation of a censored system of equations. Am J Agric Econ 81: 972–982.

3. Andreyeva T, Long MW, Brownell KD (2010) The impact of food prices on consumption: a systematic review of research on the price elasticity of demand for food. Am J Public Health 100: 216–222. doi:10.2105/AJPH.2008.151415.

4. Wang YC, Coxson P, Shen YM, Goldman L, Bibbins-Domingo K (2012) A Penny-per-ounce tax on sugar-sweetened beverages would cut health and cost burdens of diabetes. Health Aff (Millwood) 31: 199–207.

5. Bansal D, Satija A, Khandpur N, Bowen L, Kinra S, et al. (2010) Effects of migration on food consumption patterns in a sample of Indian factory workers and their families. Public Health Nutr 13: 1982–1989. doi:10.1017/S1368980010001254.

6. Ebrahim S, Kinra S, Bowen L, Andersen E, Ben-Shlomo Y, et al. (2010) The effect of rural-to-urban migration on obesity and diabetes in India: a cross-sectional study. Plos Med 7: e1000268. doi:10.1371/journal.pmed.1000268.

7. International Institute for Population Sciences (IIPS) (2007) National Family Health Survey (NFHS-3). Mumbai: IIPS.

8. World Bank (2012) World Development Indicators. Washington D.C.: IBRD.

9. Hall KD, Sacks G, Chandramohan D, Chow CC, Wang YC, et al. (2011) Quantification of the effect of energy imbalance on bodyweight. The Lancet 378: 826–837.

10. Food and Agricultural Organization (2013) FAOSTAT database. Rome: United Nations.

11. Lim SS, Gaziano TA, Gakidou E, Reddy KS, Farzadfar F, et al. (2007) Prevention of cardiovascular disease in high-risk individuals in low-income and middle-income countries: health effects and costs. Lancet 370: 2054–2062.

12. Livesey G, Taylor R, Livesey H, Liu S (2013) Is there a dose-response relation of dietary glycemic load to risk of type 2 diabetes? Meta-analysis of prospective cohort studies. Am J Clin Nutr 97: 584–596.

13. Bremer AA, Mietus-Snyder M, Lustig RH (2012) Toward a unifying hypothesis of metabolic syndrome. Pediatrics 129: 557–570. doi:10.1542/peds.2011-2912.

14. Teff KL, Grudziak J, Townsend RR, Dunn TN, Grant RW, et al. (2009) Endocrine and metabolic effects of consuming fructose- and glucose-sweetened beverages with meals in obese men and women: influence of insulin resistance on plasma triglyceride responses. J Clin Endocrinol Metab 94: 1562–1569. doi:10.1210/jc.2008-2192.

15. Schulze MB, Manson JAE, Ludwig DS, Colditz GA, Stampfer MJ, et al. (2004) Sugar-sweetened beverages, weight gain, and incidence of type 2 diabetes in young and middle-aged women. Jama J Am Med Assoc 292: 927–934.

16. Danaei G, Finucane MM, Lu Y, Singh GM, Cowan MJ, et al. (2011) National, regional, and global trends in fasting plasma glucose and diabetes prevalence since 1980: systematic analysis of health examination surveys and epidemiological studies with 370 country-years and 2.7 million participants. Lancet 378: 31–40. doi:10.1016/S0140-6736(11)60679-X.

17. World Health Organization (2012) WHO Global InfoBase. Geneva: WHO.

18. Euromonitor International (2013) Passport Global Market Information Database. New York: Euromonitor.

19. Bass FM (2004) Comments on “A New Product Growth for Model Consumer Durables The Bass Model.” Manag Sci 50: 1833–1840.

20. Akaike H (1974) A new look at the statistical model identification. Ieee Trans Autom Control 19: 716–723.

21. Hall KD, Jordan PN (2008) Modeling weight-loss maintenance to help prevent body weight regain. Am J Clin Nutr 88: 1495–1503.

# 
